# Combining Different In Vitro Bioassays to Evaluate Genotoxicity of Water-Accommodated Fractions from Petroleum Products

**DOI:** 10.3390/toxics8020045

**Published:** 2020-06-26

**Authors:** Sarah Johann, Mira Goßen, Peter A. Behnisch, Henner Hollert, Thomas-Benjamin Seiler

**Affiliations:** 1 Department of Evolutionary Ecology and Environmental Toxicology, Goethe University Frankfurt, Max-von-Laue-Str. 13, 60438 Frankfurt am Main, Germany; mira.gossen@rwth-aachen.de (M.G.); hollert@bio.uni-frankfurt.de (H.H.); 2 Department of Ecosystem Analysis, Institute for Environmental Research, RWTH Aachen University, Worringerweg 1, 52074 Aachen, Germany; 3 BioDetection Systems b.v., Science Park 406, 1098 XH Amsterdam, The Netherlands; peter.behnisch@bds.nl

**Keywords:** Ames fluctuation assay, chromosomal aberrations, crude oil, micronucleus assay, Nf2, oxidative stress, refined fuels, U2-OS, WAF, ZF-L

## Abstract

Genotoxicity assessment is of high relevance for crude and refined petroleum products, since oil compounds are known to cause DNA damage with severe consequences for aquatic biota as demonstrated in long-term monitoring studies. This study aimed at the optimization and evaluation of small-scale higher-throughput assays (Ames fluctuation, micronucleus, Nrf2-CALUX^®^) covering different mechanistic endpoints as first screening tools for genotoxicity assessment of oils. Cells were exposed to native and chemically dispersed water-accommodated fractions (WAFs) of three oil types varying in their processing degree. Independent of an exogenous metabolic activation system, WAF compounds induced neither base exchange nor frame shift mutations in bacterial strains. However, significantly increased chromosomal aberrations in zebrafish liver (ZF-L) cells were observed. Oxidative stress was indicated for some treatments and was not correlated with observed DNA damage. Application of a chemical dispersant increased the genotoxic potential rather by the increased bioavailability of dissolved and particulate oil compounds. Nonetheless, the dispersant induced a clear oxidative stress response, indicating a relevance for general toxic stress. Results showed that the combination of different in vitro assays is important for a reliable genotoxicity assessment. Especially, the ZF-L capable of active metabolism and DNA repair seems to be a promising model for WAF testing.

## 1. Introduction

Genotoxicity is an important endpoint of the (eco)toxicological risk assessment of petroleum products, since petroleum compounds have been demonstrated to cause DNA damage, with some (e.g., benzo[a]pyrene, phenanthrene) even being converted into carcinogens during xenobiotic biotransformation [1,2,3,4]. During phase I biotransformation, DNA-adducts can be formed, which might intercalate into the DNA and hence induce mutations or strand breaks [5,6]. 

Genotoxic effects are defined as deleterious actions on the genetic material affecting a cell’s integrity [7]. Genotoxicity can manifest on different levels of biological organization from gene over chromosome to complete genome and with this can have severe consequences for individuals or even populations [7,8]. Genotoxic damage is often linked to oxidative stress induction. Reactive radicals can interact with cellular macromolecules, which can lead to DNA damage [9,10]. Hence, in addition to detections of direct DNA damage, the oxidative stress potential (e.g., by means of antioxidant enzyme regulation, reactive oxygen species (ROS) production) can further contribute to the understanding of underlying adverse genotoxic effects. 

Previous laboratory and field monitoring studies have demonstrated that the contamination of the aquatic environment with crude or refined oils led to genotoxic effects in invertebrate (e.g., mussels) and vertebrate (e.g., fish) species [11,12,13]. Importantly, genotoxic damages have been reported as long-term consequences of oil contamination following large oil spills from the past decades, such as the *Haven* or *Prestige* tanker incidents [14,15,16].

In aquatic ecotoxicology, in vitro-based genotoxicity assays have successfully been established, complementing in vivo endpoints from field experiments, such as the micronucleus induction in mussel hemolymph and tissues and fish peripheral erythrocytes [17,18,19]. Major advantages of in vitro-based approaches are the reduction of animal experiments, the cost and time efficiency, and more insights into potential toxicity mechanisms by identifying molecular interactions [20]. Using a variety of cell lines, such assays can detect the potential of complex environmental samples to induce specific point or base-exchange mutations, DNA adducts, or chromosomal aberrations [21,22]. Recently, a consensus battery of effect-based methods, including genotoxicity endpoints (Ames and micronucleus assay), for water quality assessment was suggested by the EU project SOLUTIONS, which is currently processed to be implemented into the Water Framework Directive (WFD) by the NORMAN (Network of reference laboratories, research centers, and related organizations for monitoring of emerging environmental substances) network and the CIS (Common Implementation Strategy) working group of the European Commission for effect-based methods [23,24]. 

Against this background, the scope of the current study was to investigate the genotoxic potential of three different oil types using three in vitro-based bioassays, which cover different mechanistic endpoints of genotoxicity. As one of the most applied and validated genotoxicity tests, the micronucleus assay with a permanent zebrafish liver (ZF-L) cell line is aimed at detecting structural or numerical chromosomal aberrations [18,21]. Since the liver is the major organ for metabolic activity, detoxification, and homeostasis, such hepatic cell lines might particularly be useful for petroleum-related toxicity. ZF-L cells have been shown to sensitively respond to petroleum oil-relevant compounds (e.g., polycyclic aromatic hydrocarbons, PAH) on the transcriptional and protein level [25,26,27]. The Ames fluctuation assay focused on the potential of oil compounds to induce mutations in different strains of the bacterium *Salmonella typhimurium* [28]. In order to detect compounds with a pro-mutagenic character, which could be activated via vertebrate biotransformation [3], the application of a metabolic activation system from rat liver (S9) was included. Potential oxidative stress was examined by means of the Nrf2-CALUX^®^ assay, which detects the activation of the nuclear factor erythroid 2 (NFE2)-related factor 2 (Nrf2). Nrf2 is typically associated with antioxidative stress response genes [29,30,31] and, hence, is an indirect method to identify the imbalance in oxyradical production.

Optimized for the testing of water-accommodated fractions (WAFs) of different oil types, the present study addresses whether the combination of the selected bioassay battery is a useful screening tool for genotoxicity assessment of petroleum products. Since the application of a chemical dispersant is a common response strategy to combat oil spills at sea, different approaches of chemically dispersed water-accommodated fractions (CEWAFs) of oil and the dispersant itself (high-energy water-accommodated fractions, HEWAFs) were included. An additional approach using an inert oil was included to evaluate whether the physical characteristics of an oil could influence a potential toxicokinetic of a dispersant. In the context of evaluating a useful genotoxicity bioassay battery, the study addressed whether: (a) Dissolved or particulate oil fractions induce oxidative stress and/or genotoxicity, (b) a dispersant contributes to potential effects in chemically dispersed oil approaches, and (c) different oil types induce genotoxicity to a variable extent.

## 2. Materials and Methods 

### 2.1. Sample Information

In the present study, three different petroleum products were used. As a crude oil a light naphthenic North Sea crude oil (NNS, Equinor Stavanger, Norway), characterized by low viscosity, was used. Refined fuel oils were a light and low-viscous marine gas oil (MGO, Esso Norge AS, Norway) as well as a very viscous and blended heavy fuel oil (IFO 180, Polaroil, Greenland). In order to investigate the influence of chemical dispersant application on genotoxicity, the two third-generation dispersants Finasol OSR 51^®^ and 52^®^ (Total Special Fluids, Paris la Défense, France) were used [32,33]. The dispersants were selected within the framework of the EU-funded project GRACE (integrated oil spill response actions and environmental effects) [34] due to their relevance in the study region (Baltic Sea, northern Atlantic Ocean). Additionally, Miglyol 812^®^ (Caesar and Loetz GmbH, Hilden, Germany) was used as an inert oil, which is composed of medium-chain triglycerides, mainly fitting physical characteristics of the less viscous NNS.

**Figure 1:**
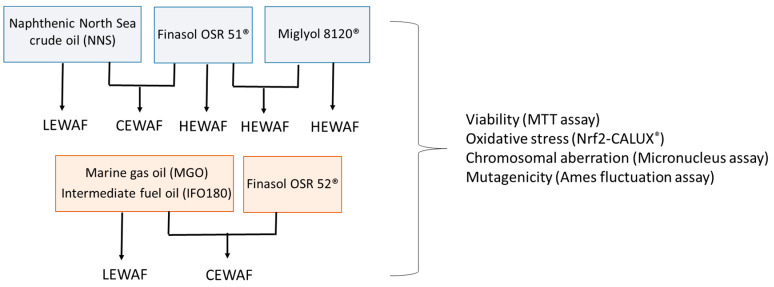
Overview of different WAF approaches and investigated endpoints. Low energy (LE-), chemically enhanced (CE-), and high-energy (HE-) WAFs of individual combinations were prepared according to Singer et al. [36]. The petroleum product types were combined with different dispersants due to oil-specific characteristics.

Similar to our previous study on the estrogenic potential of crude oil [35], the NNS crude oil and the dispersant Finasol OSR 51^®^ (representative for both Finasol dispersants in the current study) were combined in different exposure scenarios of WAFs to investigate different aspects of the petroleum product’s genotoxicity (Figure 1). First, cells were exposed to the oil alone in so-called low-energy water-accommodated fractions (LEWAFs). Second, the role of a dispersant in oil toxicity was addressed in more detail. Cells were exposed to a combination of oil and dispersant (chemically enhanced water-accommodated fractions, CEWAFs) and to the dispersant itself (high-energy water-accommodated fractions, HEWAFs). HEWAF concentrations were identical to those used for the CEWAF preparation to allow a direct comparison of potential effects. A third approach using the inert oil with and without the dispersant addressed the research question of whether an oil could also influence the toxicokinetic of a dispersant in biota and hence affect the toxicity. 

In addition to the detailed WAF approaches combined with the NNS crude oil, both refined fuel oils (MGO, IFO 180) were investigated in the LEWAF and CEWAF approaches. This aimed at identifying potential deviating genotoxic effects across different oil types. Due to project-internal recommendations for the different oil types, Finasol OSR 51^®^ was combined with the NNS crude oil, while Finasol OSR 52^®^ was combined with MGO and IFO 180.

### 2.2. Preparation of Water-Accommodated Fractions (WAFs) for Cell Exposure

WAFs were prepared in ultrapure water according to Singer et al. [36] with specific modifications [35] with respect to the study region (Baltic Sea) of the GRACE project. Briefly, WAFs were prepared in small-scale aspirator flasks (500 mL) by carefully adding oil or oil-dispersant mixtures (dispersant-to-oil-ratio, DOR = 1:10) on the water surface (at 10 °C). The LEWAF setup was carefully stirred with low energy, avoiding a vortex in the water phase. CEWAFs and HEWAFs were stirred at higher stirring speeds with 25% vortex in the water column. Resulting nominal stock concentrations were 1:50 (w/v) for LEWAFs, 1:200 (w/v) for CEWAF, and 1:2000 (w/v) for HEWAF. All WAFs were incubated at 10 °C for 40 h in the dark followed by 1 h of settling time. 

### 2.3. Chemical Analysis of Target PAHs in WAF Solutions

A set of 18 target PAHs were extracted from the LEWAF stock solutions by solid-phase micro extraction (SPME). Loaded SPME fibers were analyzed in a GC system (7890 A GC System and 5975 C inert XL MSD with Triple-Axis-Detector, Agilent Technologies Germany GmbH) according to Potter et al. [37] and as described in detail earlier [35]. Target PAHs were quantified using external and internal standards (external: S-4008-100-T, internal: S-4124-200-T, Chiron AS, Trondheim, Norway). PAHs were mainly based on priority PAHs for human health, some indicating carcinogenicity [38]. 

### 2.4. MTT Assay on Cell Viability

To exclude false negative results based on cytotoxicity, the concentration ranges of WAF dilutions resulting in normal cell viability were elaborated by using the colorimetric viability MTT assay as described in Mosmann et al. [39]. Assay procedures, including seeding, exposure, and incubation, were identical to those used for the individual cell lines (CALUX^®^, micronucleus assay). Exposure medium was removed, cells were washed with phosphate buffer saline (PBS), and yellow MTT salt (3-(4,5-Dimethylthiazol-2-yl)-2,5-diphenyltetrazolium bromide, 500 µg mL^−1^) was added. After incubation (30 min, 37 °C), the MTT solution was replaced by Dimethyl sulfoxide (DMSO) and incubated (15 min). The absorbance at 492 nm of unexposed cells was defined as 100% cell viability, while all sample dilutions were calculated relative to this viability. Results are presented in the SI, Section S1 (Figures S1 and S2).

### 2.5. Nrf2-CALUX^®^ Assay for Oxidative Stress

#### 2.5.1. Human Osteosarcoma U2-OS Cell Culture 

The U2-OS cell line, which was stably transfected with the transcription factor Nrf2 and a reporter gene construct for luciferase expression [30], was kindly provided by BioDetection Systems BV (BDS), Amsterdam, The Netherlands. Cells were cultured in 75-cm^2^ flasks according to Legler et al. [40] in a mixture of Dulbecco’s modified Eagle’s and F12 medium (1:1), supplemented with fetal calf serum (FCS, Biowest, Cholet, France), non-essential amino acids, and a penicillin-streptomycin solution. Cells were incubated at 37 °C and a humid atmosphere with 5% CO_2_. Cells were passaged regularly when reaching 90% of confluence. 

#### 2.5.2. Assay Procedure 

The Nrf2-CALUX^®^ assay was performed according to the SOP provided by BDS with specific modifications due to WAF characteristics elaborated in pretests. Cytotoxicity was excluded by pretests (MTT assay, see SI, Section S1). Material adaptions of commercial CALUX^®^ assay procedures [41,42] to WAF sample testing included the usage of glass-coated 96-well plates (WebSeal Plate+, VWR, Darmstadt, Germany) and glass plate covers (details in [35]). 3×-concentrated assay medium prepared from cell culture medium powder (Sigma, D2902) was supplemented with FCS (charcoal stripped, Biowest, Cholet, France), non-essential amino acids, and penicillin-streptomycin as described in ISO guideline no 1904-3 [43]. 

24 h after seeding cells in 96-well plates (1 × 10^5^ mL^−1^) in 1×-concentrated assay medium (diluted from 3×-concentrated medium), cells were exposed to dilution series of the reference compound curcumin (1 × 10^−8^–1 × 10^−4^ M) and treatments (LEWAF, CEWAF, HEWAF). WAF dilution series (1:2) were prepared from the 100% stock solution using sterile ultrapure water. WAF dilution series were complemented with 3×-concentrated assay medium (1:3) to guarantee an equal nutrient supply in all dilution steps. 

To stop the exposure, cells were lysed (25 mM Tris, 2 mM DTT, 2 mM CDTA, 10% glycerol, 1% TritonX^®^-100) after 24 h. Luciferase activity was measured with the application of 100 µL of luciferin substrate mixture (20 mM Tricine,1.07 mM (MgCO_3_)_4_Mg(OH)_2_5, 2.67 mM MgSO_4_ × 7 H_2_O, 0.1 mM EDTA, 1.5 mM DTT, 539 µM D-Luciferin, 5.49 mM ATP) and 100 µL reaction stop reagent (0.2 M NaOH) using a luminescence reader (Glomax 96-microplate reader, Promega, Madison, WI, USA). 

Based on luminescence data induction factors (IFs) were calculated for each dilution step of the curcumin standard and the WAF treatments by normalizing each luminescence value to the luminescence of the background of the standard in order to quantify the measured response. A concentration-response curve was established with the IF values (4 parameters non-linear regression with a variable slope) using Prism 6 (GraphPad, v. 6, San Diego, CA, USA). Within this concentration-response fit, the concentration of curcumin and the treatment that resulted in an IF = 1.5 were calculated. The specific IF (1.5) was used due to non-cytotoxic and stable response within this range. Finally, the specific Nrf2 activity was calculated by dividing the sample concentration by the standard concentration at IF = 1.5. 

### 2.6. Micronucleus Assay on Chromosomal Aberration 

#### 2.6.1. Zebrafish Liver (ZF-L) Cell Culture 

The permanent ZF-L cell line [44] was cultured in L15 medium (Leibovitz, with L-glutamine, Sigma Aldrich, L4386), supplemented with 10% fetal calf serum (FCS, Biowest, Cholet, France) in 75-cm^2^ flasks at 28 °C. Cells were passaged regularly when reaching 90% of confluence. 

#### 2.6.2. Assay Procedure 

The micronucleus assay was performed according to the ISO guideline 21427-2 [45] and Bluhm and Heger et al. [46] with modifications regarding ZF-L growth (doubling time) and WAF testing. Exposure dilutions of the WAF were elaborated in pretests (see MTT results in SI) in order to avoid cytotoxicity. The 3×-concentrated assay medium was prepared from L15-powder and supplemented with FCS (charcoal stripped, Biowest, Cholet, France) and penicillin-streptomycin. 

Cell suspension (in 1×-concentrated assay medium, diluted from 3×-concentrated medium) at a density of 5× 10^4^ was seeded in sterile small glass petri dishes (40 mm, VWR, Darmstadt, Germany) containing sterile cover slips (20 × 20 mm, VWR, Darmstadt, Germany). After 24 h, cells were exposed to dilution series of WAF treatments in duplicates. To evaluate the test validity, a negative control (assay medium only), a positive control (4-Nitroquinoline 1-oxide, exposure concentration 6.22 × 10^−8^ M, stock in DMSO), and a solvent control (0.1% DMSO) were included. After 48 h of exposure, the cover slips with attached cell layers were fixed with MeOH: acetic acid (4:1, each for 5 min), air dried, and finally glued onto glass slides. Microscopy slides were stained using acridine orange dye. An Eclipse 50i epifluorescence microscope (Nikon Instruments, Düsseldorf, Germany) with 40 × magnification was used to generate pictures, in which micronucleated cells were identified according to the following criteria (ISO 21427-2): (a) The maximum size of a micronucleus was one-third of the main nucleus, (b) micronuclei had the same staining intensity as normal nuclei, (c) micronuclei were clearly separated from the nucleus, and (d) only cells with clear plasmatic outlines were observed. A total of 2000 cells per treatment were evaluated for micronuclei formation. Statistical analysis for each replicate was done by the Chi^2^ test with Yates correction using SigmaStat 12.5 (Systat GmbH, 2007). Validity criteria were met when in negative and solvent controls not more than 3% of counted cells contained micronuclei and the positive control induced a significant increase in micronucleated cells in the statistical Chi^2^ test. 

### 2.7. Ames Fluctuation Assay on Mutagenicity 

#### 2.7.1. *Salmonella Typhimurium* Bacterial Strain 

The two tester strains *S. typhimurium* TA98 and TA100 (TrinovaBiochem GmbH, Giessen, Germany) were used, which indicate the potential to induce frame shift and base pair exchanges mutations. Sub-aliquots of one batch (stored at −80 °C) were thawed for each experiment.

#### 2.7.2. Assay Procedure 

The Ames fluctuation assay was performed according to ISO 11,350 [47] with water samples described in Reifferscheid et al. [48] and WAF sample testing detailed in Bluhm and Heger et al. [46]. The metabolic activation system S9 obtained from rat liver (induced with β-naphthoflavone/phenobarbital, Envigo, Ettlingen, Germany) was added to exposure solutions, in order to detect a possible pre-mutagenic character of sample compounds. Hence, bacteria were exposed to LEWAF, CEWAF and HEWAF solutions with and without S9. Cytotoxic concentration ranges of each sample excluded in pretests within the normal fluctuation assay procedure (see SI). 

Bacterial overnight cultures of both tester strains were prepared before testing and incubated at 37 °C and 150 rpm for 9.75 h in an Innova−40 incubation shaker (New Brunswick, Scientific, Edison, NJ, USA).Overnight cultures were adjusted to a certain cell density (1800 FAU for TA98, 450 FAU for TA100), and exposure medium, and, if needed, supplemented S9 fraction was added in a sterile 24-well glass plate (Hellma Analytics, Müllheim, Germany). Besides treatment concentrations negative and strain-specific positive controls were tested for validity evaluation (10 mg L^−1^ 4-nitro-o-phenylenediamine for TA98 without S9, 0.1 mg L^−1^ 2-aminoanthracenefor TA98 with S9, 0.25 mg L^−1^ nitrofurantoin for TA100 without S9, 0.4 mg L^−1^ 2-aminoanthracene for TA100 with S9). After 100 min of incubation (37 °C, 150 rpm), cell suspension was transferred to a 384-well plat, with 16 replicated wells per treatment concentration, followed by 48 h incubation at 37 °C. Spontaneous or exposure induced bacterial revertant formation were detected by color change of the pH-sensitive reversion indicator medium. A test was counted as valid with negative control leading to less than 10 revertant wells positive control inducing more than 25 revertant wells. Revertant wells were counted, followed by statistical analysis using, the software ToxRat (ToxRat Solutions GmbH, Alsdorf, Germany) was used. Within this, a arcsine transformation of the reversion rate was performed and homoscedacity and normal distribution was verified. Williams multiple t-test was used to determine significant differences of the treatments from the control. 

## 3. Results

### 3.1. Target PAHs in LEWAF Stock Solutions

From the 18 target PAHs, LEWAF stocks of all three oil types mainly contained lower molecular weight PAHs of 2–3 rings (Table 1). The predominant PAHs were naphthalene (200–400 µg L^−1^), followed by fluorene and phenanthrene (6–9 µg L^−1^). Furthermore, 4–6 ring PAHs, e.g., benzo[a]pyrene, dibenz[a,h]anthracene, and dibenzo[a,e]pyrene, were detected either in an ng L^−1^ range or below the limits of quantification or detection (LOQ; LOD). LEWAFs from the NNS crude oil contained higher concentrations of target PAHs (Σ PAH = 443.2 µg L^−1^) than both marine fuels (Σ PAH = 244.5 (MGO) and 235.99 (IFO 180) µg L^−1^). The discrepancy was mainly based on the concentration of naphthalene. Without this 2-ring PAH, all three oil types contained PAHs in a comparable range (Σ PAH = 11.7 (NNS), 15.0 (MGO), and 16.2 (IFO 180) µg L^−1^). 

### 3.2. Oxidative Stress Response in U2-OS Cells Using the Nrf2-CALUX^®^ Bioassay

**Figure 2:**
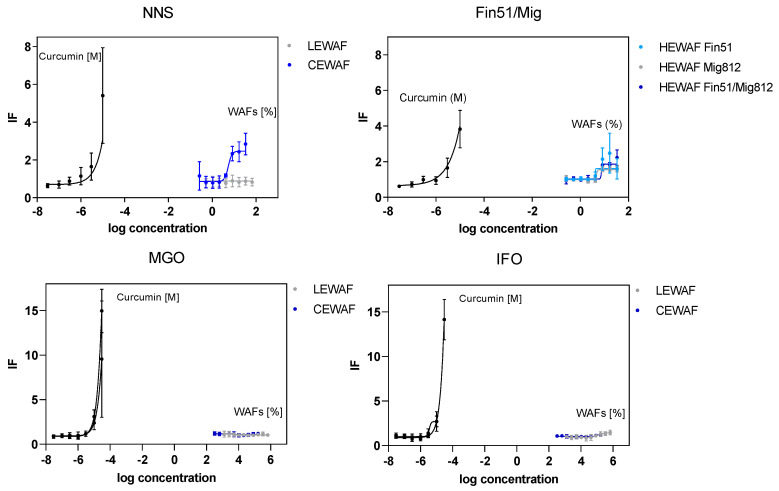
Induction of oxidative stress after exposure to WAF dilutions of petroleum products, dispersant, and inert oil in the Nrf2-CALUX^®^ assay. Approaches include crude (NNS) and refined petroleum products (MGO, IFO 180), the dispersant Finasol OSR 51 (Fin51), and the inert oil Miglyol (Mig812). Based on luminescence data, the induction factors (IFs) were calculated as relative values to the background of the Curcumin calibration series. Symbols and error bars represent the mean IF of 3–4 independent experiments with standard deviation. HEWAFs were prepared using corresponding amounts to CEWAF approaches. A non-linear regression model with variable slope was used to fit the concentration–response curves in Prism 6 (GraphPad v 6).

A limited set of WAF treatments did activate the transcription factor Nrf2, indicating the potential for oxidative stress in human osteosarcoma cells. The chemically dispersed crude oil (NNS CEWAF) as well as the dispersant itself (HEWAF Fin51), inert oil (HEWAF Mig812), and dispersed inert oil (HEWAF Fin 51/Mig812) showed a clear concentration-related increase in Nrf2 activity (Figure 2). Within this, almost identical concentration–response curves were observed for CEWAF NNS and HEWAF Fin51, while the inert oil (HEWAF Mig812) as well as the chemically dispersed inert oil (HEWAF Fin51/Mig812) showed less Nrf2 induction. In contrast, MGO constituents did not induce the Nrf2. For cells exposed to the IFO 180 WAFs, only the LEWAF induced a slight increase of Nrf2 activity at the highest test concentration.

The specific activity, expressed as the relative activity of ng Curcumin µL^−1^ sample, was calculated in order to allow a better comparability to other studies. With the exception of LEWAF IFO 180 (9 ng Curc µL^−1^), the oxidative stress was not quantifiable for LEWAFs of the remaining oil types (Table 2). Additionally, the application of a chemical dispersant led to activities above the quantification limits only for the NNS crude oil (19.2 ng Curc µL^−1^). Even higher mean specific activities were calculated for the treatments HEWAF Fin51 (21.0 ng Curc µL^−1^) and HEWAF Fin51/Mig812 (32.1 ng Curc µL^−1^), respectively. The HEWAF Fin51/Mig812 response was characterized by a high standard deviation caused by one out of three independent replicates, which might relativize this clear Nrf2 induction.

### 3.3. Chromosomal Aberrations in ZF-L Cells Using the Micronucleus Assay

**Figure 3:**
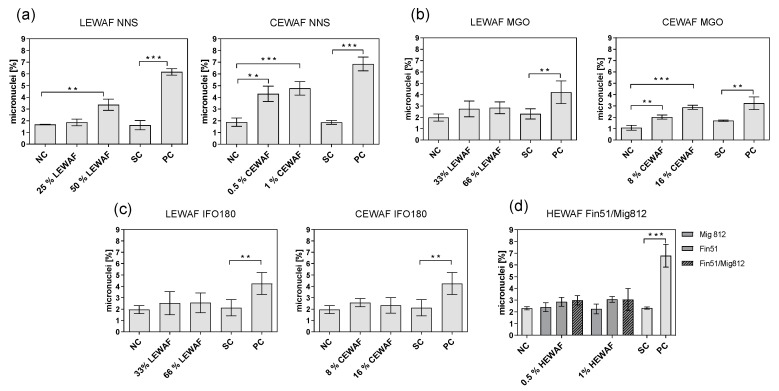
Micronucleus induction in ZF-L cells exposed to WAF dilutions of crude oil (NNS, **a**) and refined petroleum products (MGO, **b**, IFO 180, **c**) as well as dispersant combinations (HEWAF, **d**). The dispersant Finasol OSR 51^®^ was tested in corresponding concentrations to the NNS CEWAF treatment. Bars represent the mean percentage of micronucleated cells (out of 2000 counted cells) with error bars indicating the standard deviation (*n* = 3–4). Negative- (NC), solvent- (SC, 0.1% DMSO) and positive (PC, 4-Nitrochinolin-1-oxide, 0.1% DMSO) controls were included. Chi^2^ test with Yates correction was used for statistical analysis. Asterisks indicate significantly higher micronuclei induction compared to controls (** *p* < 0.01, *** *p* < 0.001). All data met the validity criteria (NC micronuclei < 3%, PC significant micronuclei induction) defined by the ISO guideline 2147-2 on genotoxicity [45].

Both LEWAF and CEWAF of the NNS crude oil induced significantly increased micronuclei formation compared to the unexposed control (Figure 3a). Within this, the chemically dispersed oil exposure (IF = 2.61 ± 0.73, see Table 3) resulted in more chromosomal aberrations than the untreated oil exposure (IF = 2.01 ± 0.29). Deviating from the NNS crude oil exposure, neither MGO (Figure 3b) nor IFO 180 (Figure 3c) showed a significantly increased genotoxic potential in cells exposed to WAFs, with micronuclei frequencies comparable to the untreated control (IF = 1.3–1.4). One exception was the chemically dispersed MGO with significantly increased micronucleated cells compared to the control (IF CEWAF MGO = 2.74 ± 0.54). Hence, a trend of a decreased genotoxic potential across the oil types was observed (LEWAF: NNS > MGO = IFO 180, CEWAF: NNS > MGO > IFO 180).

In order to evaluate the influence of the dispersant on the elevated micronuclei formation of CEWAF compared to LEWAF exposure, the micronucleus assay was performed with HEWAF of Finasol OSR 51^®^ in concentrations allowing a direct comparison to the CEWAF results. The Finasol OSR 51 HEWAF^®^ treatment resulted in micronuclei induction that was slightly but not significantly increased compared to the untreated control (Figure 3d), which was 2-fold below the micronuclei rates induced by the CEWAF treatment. Additionally, no significant micronucleus formation was observed for the inert oil and the dispersed inert oil.

### 3.4. Mutagenicity Using the Ames Fluctuation Assay

No significant increase in the revertant formation compared to the negative control was observed for both tester strains (TA 98 and TA 100) across all oil types and WAF types. Even the application of the S9 fraction obtained from rat livers to detect a pre-mutagenic character, which could be activated by the liver enzymes, did not convert the WAF components into DNA-intercalating compounds. Detailed results of the revertant formation can be found in the SI (Table S1).

## 4. Discussion

### 4.1. Oxidative Stress

The current study is the first to our knowledge using the Nrf2-CALUX^®^ bioassay in the context of petroleum oil WAF testing. This assay is well-established in the field of fresh and drinking water quality assessment [49,50]. Of the limited set of target PAHs that had already been investigated in the Nrf2-CALUX^®^ assay as individual compounds (dissolved in DMSO), neither phenanthrene, which is of high relevance in the current LEWAFs, nor pyrene, benzo[a]pyrene, and dibenzo[a,h]pyrene had induced the transcription factor Nrf2 [51]. The fact that some petroleum WAFs of the current study (CEWAF NNS, LEWAF IFO 180) did interact with this receptor in a concentration-related and quantifiable manner might be related to other WAF constituents not characterized by the conducted chemical analysis of target PAHs. WAFs consist of thousands of compounds acting as a complex mixture [36] with hardly predictable effects on biota.

In general, PAHs, crude oils, and produced waters are well known for their potential to induce oxidative stress through increased formation of reactive oxygen species (ROS) in fish tissues [52,53] as well as primary and permanent cells [54,55]. Additionally, petroleum WAFs and relevant compounds (PAHs) have induced strong alterations of antioxidant enzyme activities like catalase or superoxide dismutase [53,56,57,58]. As previously discussed for other mechanism-specific endpoints using in vitro-based assays [35,42], it has to be considered that the U2-OS cells do have limited capability for metabolization [51]. Thus, the toxicity of many petroleum constituents like phenanthrene or benzo[a]pyrene, which occurs after bioactivation during xenobiotic biotransformation [5,6], were not addressed with this transgenic cell line. All referred in vitro-based assays detecting strong oxidative stress response used (hepatic) cell lines that are capable of active metabolization. Hence, future experiments focusing on petroleum-induced toxicity in the U2-OS cell line should consider the simulation of a vertebrate xenobiotic biotransformation system (e.g., S9 fraction obtained from rat livers) [41,59].

### 4.2. Genotoxicity

Current results of significantly elevated micronucleus frequencies in ZF-L cells indicate a genotoxic potential of dissolved and particulate WAF compounds. The application of the in vitro-based micronucleus assay, particularly using the ZF-L cell line, for petroleum genotoxicity evaluation is rather scarce. Nonetheless, Lachner et al. [60] exposed ZF-L cells to gasoline WAFs focusing on different genotoxic endpoints, including the antioxidant capacity and DNA damage (Comet assay). The authors found a strong genotoxic potential already after short-term exposure, which in the current study was only observed after chemical dispersion of the light fuel oil (MGO CEWAF). Differences between the two studies might be related to higher exposure concentrations that have been used by Lachner et al. [60]. In general, a good correlation between the two most frequently used genotoxicity assays (Comet, micronucleus assay) has been reported, which has particularly been indicated for PAH-containing extracts [61] or fossil fuel WAFs [60,62].

In contrast to the significant chromosomal aberrations, the present results indicate that the petroleum products do not cause frame shift or base exchange mutations in the *Salmonella* strains TA 98 and TA 100. These findings are in compliance with previous studies focusing on the mutagenicity of complex WAFs from crude oils and refined petroleum products, which also did not reveal any induction of mutations in different tester strains [46,63,64,65].

This observation might be explained by the general low genotoxicity of dominant low molecular weight PAHs from the current LEWAFs (naphthalene, fluorene, and phenanthrene). Tested as individual compounds in the Ames assays, several studies found those compounds to be inactive independent of the application of the vertebrate xenobiotic metabolizing system (S9) or tester strain [66,67,68,69]. Additionally, Schreiner et al. [69] concluded a low genotoxic potential for naphthalene after screening a large dataset on different genotoxic endpoints. However, especially higher molecular weight PAHs, such as benzo[a]pyrene, which was detected in concentrations varying from 80 to 170 ng L^−1^ in the present LEWAFs, have been reported to be active in both the *Salmonella* - [65,67] and the micronucleus assay [70].

Importantly, the genotoxicity of complex mixtures like oil WAFs can differ from observations for individual compounds. A recent study on chromosomal aberrations showed that the potential of PAH mixtures to induce micronuclei remains unpredictable already from binary mixtures on [71]. Furthermore, secondary effects of the exposure have to be considered. Naphthalene, for example, neither inducing mutations nor chromosomal aberrations or DNA adducts, has been shown to cause secondary damage to DNA [72]. Such secondary genotoxicity might also lead to severe consequences for an exposed organism. The complexity of mixture toxicity, including additive, synergistic, or antagonistic effects, as well as secondary toxicity emphasizes the importance of individual toxicity profiles of unique oil types for a reliable risk assessment.

### 4.3. Comparison of Different Oil Types and the Influence of Chemical Dispersant Application

Due to the overall lack of response, the mutagenic potential was not included in this discussion. Results of the Nrf2-CALUX^®^ assay neither allowed a clear conclusion to be drawn about the oil type-specific intensity of oxidative stress nor about patterns regarding initial and chemically dispersed oil. In contrast, clear differences in micronuclei frequencies were observed across the different oil types, with the NNS crude oil inducing the most chromosomal damage. Based on the chemical analysis of target PAHs, the decrease in micronucleus frequencies correlated mainly with a sharp decline of the naphthalene concentrations up to 50% from NNS to refined fuels (MGO, IFO 180). Remaining higher molecular weight target PAHs were detected in a comparable concentration range or even marginally increased in the refined fuels. However, the chemical analysis focused on a limited set of target PAHs, representing only a small portion of the complex WAFs [36] that potentially do have a limited contribution to mixture toxicity. In this context, PAH derivates of, e.g., phenanthrene have been found to induce significantly increased micronuclei rates in permanent cell lines [73], while the parent compound did not [70]. The limited explanation of WAF-induced biological effects by chemical profiling (e.g., via total petroleum hydrocarbon analysis) has already been observed previously [74,75]. In addition, it has to be considered that the WAF concentrations change throughout the exposure, even though absorption is expected to be reduced in the glass-coated well plates used.

The present experimental setup allowed more insights into the potential impact of a chemical dispersant on the genotoxicity of oil WAFs. First, the application of chemical dispersants increased the rate of chromosomal aberrations based on the increased bioavailability of dissolved and particulate oil compounds [74,76,77,78], since the dispersant alone led to micronuclei formation comparable to the unexposed control. The exception that the application of a chemical dispersant to the IFO 180 did not significantly increase the chromosomal aberrations might be related to the physical oil properties. Very viscous oils, like IFO 180, are less easy to disperse compared to lighter products, such as the NNS and MGO [79,80]. Second, the dispersant alone did induce oxidative stress to an extent comparable with corresponding chemically dispersed oil. Additionally, the inert oil and chemically dispersed inert oil treatments resulted in quantifiable Nrf2 activation, indicating oxidative stress induction. Hence, the dispersant, being a complex mixture of surfactants and hydrocarbon solvents, has the potential to induce general toxic stress independent of a mechanism-specific toxicity. From the approach combining the inert oil and the dispersant, it was further indicated that the toxicokinetic of a dispersant is rather not impacted by the presence of an oil. The current dispersant-related observations emphasize that the role of a chemical dispersant in CEWAF toxicity should not be excluded per se. However, the different modes of action of dispersant toxicity, either via narcosis or other regulatory pathways, need to be addressed in more detail in future research.

### 4.4. One Assay Is Not Enough: Combining In Vitro-Based Methods for Genotoxicity Assessment of Petroleum Products

As shown in the current study, complex samples, such as petroleum WAFs, can initiate genotoxicity in one but not in all bioassays, which has already been observed in previous studies working with WAF exposure [46]. Hence, it is important to combine different bioassays that contribute to the understanding of different modes of action of genotoxicity. As reviewed by Kirkland et al. [2,81], the combination of two to three test systems, involving the Ames and micronucleus assay, showed a high sensitivity in the identification of rodent carcinogens and in vivo genotoxicants by in vitro-based methods. Results of the present study recommend this combinatory approach not only for individual compounds but also for complex water samples.

Most studies addressing the genotoxicity in aquatic biota exposed to petroleum samples have focused on in vivo micronucleus frequencies in peripheral erythrocytes of fish or mussel tissues [11,13]. The present results also suggest the in vitro-based micronucleus assay as a valuable and sensitive screening tool in oil genotoxicity assessment when applied considering important experimental aspects as well as interpretation limitations. The Ames fluctuation assays did not detect mutagenicity in the current study. However, its value for oil genotoxicity assessment cannot be excluded based on the current findings. Several tester strains with different mutation types exist, which detect a variety of mutation types and thus might result in significant responses [82,83]. The Ames assay has been proven as a sensitive method to detect mutagenic and potentially carcinogenic compounds [84]. Though the current study was not able to show a correlation between the induction of oxidative stress and DNA damage, bioassays on oxidative stress can provide useful information for toxicity assessment and should be included in a genotoxicity battery.

As indicated above, limitations have to be considered for a reliable effect interpretation. The tendency of misleading (“false”) positive responses in in vitro assays poses a challenge for the extrapolation of results to real scenarios. In particular, misleading positive in vitro micronuclei frequencies of non-genotoxic compounds have been observed in several cell lines [85]. However, the false positive genotoxicity has mainly been be associated with cells lacking metabolic activation, impaired p53 function, and altered DNA repair capacity [85]. Thus, the selection of a useful cell line is of critical importance [86]. Recently, the capability of ZF-L cells for different DNA repair mechanisms similar to primary hepatocytes has been demonstrated [87]. Hence, in addition to a metabolic capacity, which is of high relevance for petroleum constituents’ genotoxicity, the ZF-L cell line seems a valuable model in oil toxicity assessment. A further reason for false positive results in the micronucleus assay is scoring artefacts related to apoptosis or necrosis as micronuclei, which potentially occur due to high cytotoxic effects [88]. However, such artefacts can be reduced by careful elaboration of non-cytotoxic exposure concentrations in pretesting, like applied in the present study.

In the context of extrapolating in vitro-based findings, it should further be considered that not every cellular event manifests on higher levels of biological organization. Thus, the screening tools can be interpreted as part of a precautionary principle for a protective risk assessment.

## 5. Conclusions

The present study aimed at combining different higher-throughput small-scale assays to assess the genotoxic potential of petroleum WAF constituents. Optimized for oil WAF testing, the results indicate that dissolved and particulate oil constituents have the potential to induce genotoxicity. It was found that the combination of endpoints is important to cover different mechanisms of genotoxicity. In this respect, future research could further optimize a potential bioassay battery of in vitro-based methods to identify DNA-damaging complex mixtures.

The dispersant rather did not contribute to chromosomal aberrations but induced oxidative stress in exposed cells. This observation stresses the importance of considering its impact for effect interpretation. The aspect of a general toxic stress should not be excluded from the oil response discussion simply because dispersants mainly increase the bioavailability of oil compounds, which needs to be addressed in future research.

The results further emphasize the importance of including biotransformation capacities for genotoxicity assessment of oil samples in in vitro assays. The application of a vertebrate metabolic enzyme mixture can only partially reproduce potential bioactivation. Using cells capable of active metabolism and DNA repair should be preferred.

It has to be considered that the present study addressed the evaluation of initial screening tools for genotoxicity. In order to ecotoxicologically characterize the genotoxic potential of oil samples, effects on higher biological organization levels should be implemented. Furthermore, long-term exposure scenarios with much lower exposure concentrations compared to the present study would cover more environmentally realistic and relevant conditions.

## Figures and Tables

**Table 1:** Target PAHs in LEWAF stocks (1:50) for cell exposure. LEWAF stocks were prepared in double-deionized water. PAHs were extracted after 40 h of mixing at 10 °C followed by 1 h of settling time using solid-phase micro extraction (SPME) for 2 h and analyzed using GC-MS. Results of the chemical analysis of NNS crude oil can be found in Johann et al. (2020) Table S1 in the SI. N.d. = not detected (below limits of quantification or limits of detection).

Target Compound	MGO (µg L^−1^)	IFO180 [µg L^−1^]
Naphthalene	229.52	219.80
Fluorene	6.10	4.64
Phenanthrene	7.33	8.77
Anthracene	n.d.	0.83
Fluoranthene	0.12	0.12
Pyrene	0.25	0.39
11h-benzo[a]fluorene	0.34	0.34
11h-benzo[b]fluorene	0.21	0.24
Benzo[a]anthracene	0.10	0.13
Chrysene	0.18	0.34
Benzo[b]fluoranthene	0.08	0.09
Benzo[k]fluoranthene	0.11	0.10
Benzo[a]pyrene	0.08	0.11
Benzo[e]pyrene	0.10	0.09
Indeno[1,2,3 cd]pyrene	n.d.	n.d.
Dibenz[a,h]anthracene	n.d.	n.d.
Benzo[ghi]perylene	n.d.	n.d.
Dibenzo[a,e]pyrene	n.d.	n.d.
Σ PAHs	244.50	235.99

**Table 2:** Calculated specific activity of crude oil (NNS) and refined petroleum product (MGO, IFO 180) WAFs in the Nrf2-CALUX^®^ assay for oxidative stress. Specific activity was calculated based on sample and curcumin (reference) concentrations, resulting in an induction factor (IF) of 1.5 corrected for the reference background and fitted with non-linear regression in Prism 6 (GraphPad. In the case when the specific activity was below the limit of quantification (LOQ), the mean LOQ was added in an additional column (*n* = 3–4).

Treatment	Mean Specific Activity (ng Curc. µL^−1^ Sample)	SD	LOQ (ng Curc. µL^−1^ Sample)	SD
LEWAF NNS	<LOQ		2.0	0.7
CEWAF NNS	19.2	10.6		
LEWAF MGO	<LOQ		2.7	1.1
CEWAF MGO	<LOQ		8.2	1.5
LEWAF IFO 180	9.0	7.0		
CEWAF IFO 180	<LOQ		9.1	0.5
HEWAF Fin 51	21.0	8.4		
HEWAF Mig 812	20.0	13.2		
HEWAF Fin51/Mig812	32.1	33.7		

**Table 3:** Calculated induction factors (IFs) of micronuclei formation in ZF-L cells exposed to WAF dilutions of crude oil (NNS) and refined petroleum products (MGO, IFO 180) as well as dispersant combinations (HEWAF). Mean IFs and standard deviations (SDs) were calculated as percentage micronuclei induction relative to an unexposed control of independent biological replicates (*n* = 3–4).

Treatment	LEWAF (% of Stock)	IF Mean	SD	CEWAF (% of Stock)	IF Mean	SD
NNS	50	2.01	0.29	1	2.61	0.73
	25	1.11	0.16	0.5	2.37	0.79
MGO	66	1.35	0.22	16	2.74	0.54
	33	1.42	0.42	8	1.90	0.24
IFO 180	66	1.32	0.37	16	1.25	0.51
	33	1.30	0.43	8	1.35	0.29
Fin51				1	1.33	0.09
				0.5	1.24	0.12
Mig 812				1	0.97	0.14
				0.5	1.04	0.14
Fin51/Mig812				1	1.32	0.38
				0.5	1.30	0.20

## Supplementary Materials

The following are available online at https://www.mdpi.com/2305-6304/8/2/45/s1, Figure S1: Relative viability of U2-OS cells exposed to WAF dilutions from refined petroleum products (MGO, IFO 180) in the MTT bioassay, Figure S2: Relative viability of ZF-L cells exposed to WAF dilutions from the NNS crude oil and the refined petroleum products (MGO, IFO 180) in the MTT bioassay, Table S1: Mean number of revertants at highest exposure concentration and corresponding mean negative controls (NC) in the Ames fluctuation assay with standard deviation (SD).
