# Supplementary material for: Combining Different In Vitro Bioassays to Evaluate Genotoxicity of Water-Accommodated Fractions from Petroleum Products
Source: Toxics. 2020 Jun 26;8(2):45. doi: 10.3390/toxics8020045 (PMC7355774; doi:10.3390/toxics8020045)
Supplement: Supplementary file 1 [file toxics-08-00045-s001.pdf]

Supplementary Materials: Combining Different In Vitro Bioassays to Evaluate Genotoxicity of Water-Accommodated Fractions from Petroleum Products

Sarah Johann, Mira Goßen, Peter A. Behnisch, Henner Hollert and Thomas-Benjamin Seiler

1. MTT Assay for Cell Viability

1.1. Viability of U2-OS Cells

The viability of U2-OS cells exposed to NNS crude oil LEWAF, and CEWAF has been presented previously [1]. Comparable to NNS crude oil both fossil fuels (MGO, IFO 180) induced stronger toxicity in dispersed oil (CEWAF) compared to native oil (LEWAF) exposure (**Figure S1**). While for the LEWAF treatments already the highest concentrations resulted in a cell viability comparable to the untreated control, the CEWAF exposure induced a toxicity with a normalized cell viability below 80% in the 2 highest exposure concentrations. CEWAF dilutions from 16.6% of stock and below did not induce critical cytotoxicity.

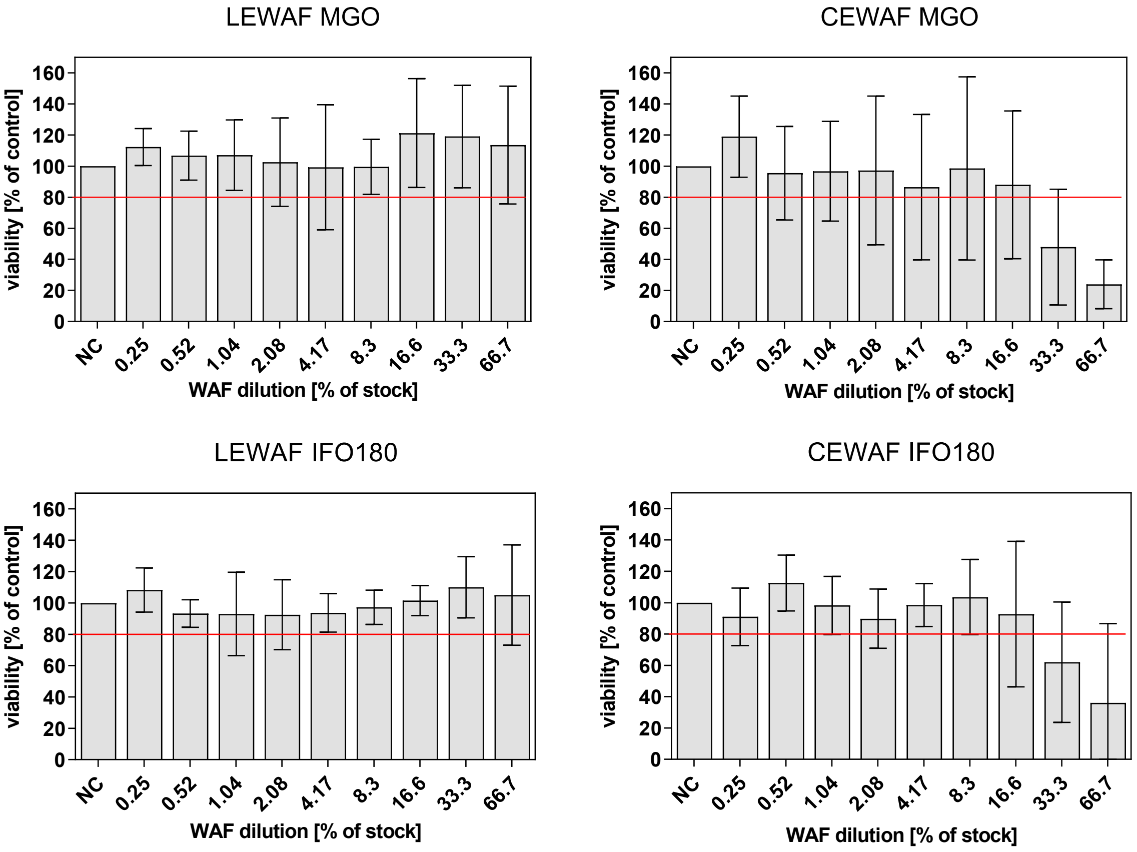

Figure S1. Relative viability of U2-OS cells exposed to WAF dilutions from refined petroleum products (MGO, IFO180) in the MTT bioassay. Cytotoxic effects of sample concentrations were normalized to the negative control (100% viability). Bars represent mean values of 3–4 independent replicates. Error bars denote the standard deviation and the red line illustrates the critical threshold for normal cell viability (80%).

1.2. Viability of ZF-L Cells

Comparable to U2-OS cells, ZF-L cells showed a trend of more cytotoxic effects caused by the CEWAF compared to the LEWAF treatments with the exception of NNS crude oil LEWAF. This treatment resulted in a clear cytotoxicity for the highest exposure concentration above the critical threshold of 80 cell viability at the highest exposure concentration (66.6% of stock).

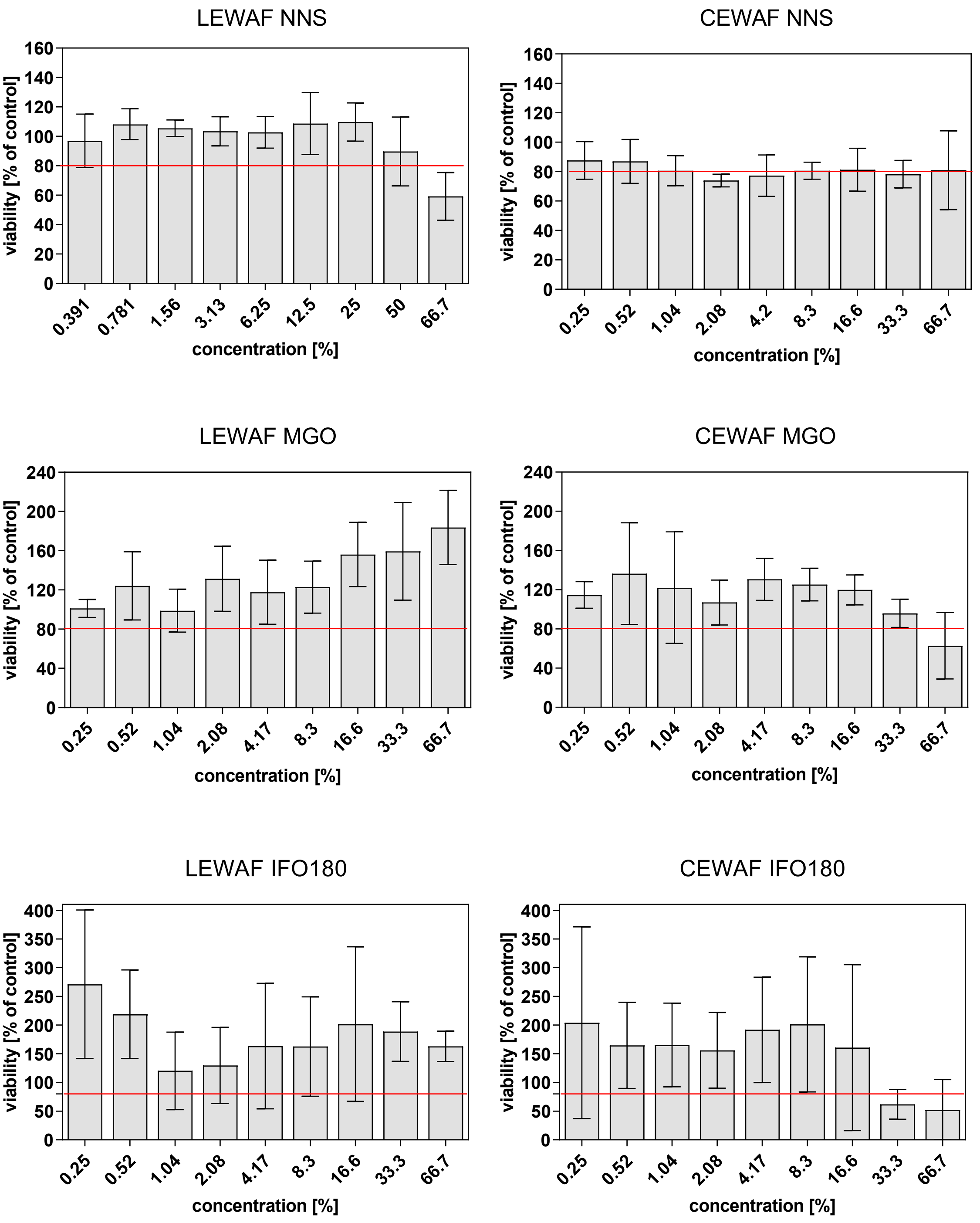

Figure S2. Relative viability of ZF-L cells exposed to WAF dilutions from the NNS crude oil and the refined petroleum products (MGO, IFO180) in the MTT bioassay. Cytotoxic effects of sample concentrations were normalized to the negative control (100% viability). Bars represent mean values of 3–4 independent replicates. Error bars denote the standard deviation and the red line illustrates the critical threshold for normal cell viability (80%).

Overall, no clear trend of increased sensitivity of one cell line over the other could be observed. Based on the cell viability results the exposure solutions for the further endpoints in this chapter varied between 25–66 % of stock (LEWAF) and 0.5–16% of stock (CEWAF).

2. Ames Fluctuation Assay for Mutagenicity

2.1. Viability Examination during Ames Fluctuation Assay Procedure with TA 98

The cytotoxicity of all WAF samples was investigated during the normal Ames fluctuation assay procedure using the tester strain TA98. Measuring the optical density before and after the short term incubation and calculating the cell growth rate indicated cell viability. The growth rate was normalized to an untreated control to calculate cytotoxicity. Concentrations resulting in cytotoxicity of 50% or higher were excluded for the mutagenicity detection.

With the exception of NNS crude oil CEWAF, all treatments did not influence normal bacterial growth and hence their mutagenic potential was tested from the undiluted stock. The maximum exposure concentration of the NNS CEWAF was 25% of stock (within test = 20% due to dilution effect based on medium application).

Table S1. Mean number of revertants at highest exposure concentration and corresponding mean negative controls (NC) in the Ames fluctuation assay with standard deviation (SD). $n = 3$. All data met the validity criteria according to the ISO guideline 11350 (ISO 2012) as detailed in Reifferscheid et al. [2]. NC was included to show baseline mutagenicity varying between different tester strains and the application of the rat liver homogenate (S9). No statistically significant increase in mutagenicity was observed across all treatments (ToxRat software (Alsdorf, Germany), using Williams multiple *t*-test).

Treatment	Max. exposure concentration [% of stock]	Mean # of revertants (SD) at max. exposure concentration							
		WAF 98 +S9	NC 98 +S9	WAF 98 -S9	NC 98 -S9	WAF 100 +S9	NC 100 +S9	WAF 100 -S9	NC 100 -S9
**NNS LEWAF**	80	1.6 (2.1)	1.6 (2.1)	4.3 (3.1)	2 (2.0)	2 (1.7)	1 (0)	5 (2.7)	7 (2.0)
**NNS CEWAF**	20	3.3 (2.1)	0.3 (0.6)	3.3 (3.5)	4 (5.13)	2.6 (1.5)	1.6 (0.6)	7.6 (5.5)	2 (2.6)
**MGO LEWAF**	80	1.3 (1.5)	0 (0)	0.6 (0.6)	1 (1.0)	2.3 (1.5)	3 (1.7)	7.7 (5.5)	6 (4.4)
**MGO CEWAF**	80	0.6 (0.6)	1 (1.0)	0.6 (0.6)	1 (1.0)	0.6 (1.2)	2.6 (1.2)	1.6 (2.1)	7.6 (1.5)
**IFO LEWAF**	80	2.6 (1.5)	0.3 (0.6)	1.3 (2.3)	1.3 (1.2)	1.6 (1.2)	2 (2.7)	8.3 (1.5)	5 (3.6)
**IFO CEWAF**	80	1.3 (0.6)	1 (1.0)	2 (1.0)	0.6 (0.6)	4 (2.0)	2.6 (3.8)	2.3 (2.6)	6.6 (1.5)
**Fin51 HEWAF**	80	0.6 (0.6)	0.3 (0.6)	2 (2.0)	3.6 (3.2)	5 (3.0)	5.6 (5.5)	2.6 (3.1)	4.3 (1.2)
**Mig812 HEWAF**	80	0 (0)	0.6 (1.2)	1 (0.0)	3 (5.2)	2 (1.0)	2 (1.0)	5.6 (2.5)	2.6 (0.6)
**Fin51/Mig812 HEWAF**	80	0 (0)	0.3 (0.6)	3.6 (5.5)	2.6 (2.1)	3.3 (2.1)	3.6 (3.1)	1 (1.0)	2 (1.0)

Reference

1. Johann, S.; Esser, M.; Nüßer, L.; Altin, D.; Hollert, H.; Seiler, T.-B. Receptor-mediated estrogenicity of native and chemically dispersed crude oil determined using adapted microscale reporter gene assays. *Environ. Int.* **2020**, *134*, 105320, doi:10.1016/j.envint.2019.105320.
2. Reifferscheid, G.; Maes, H.M.; Allner, B.; Badurova, J.; Belkin, S.; Bluhm, K.; Brauer, F.; Bressling, J.; Domeneghetti, S.; Elad, T.; et al. International round-robin study on the Ames fluctuation test. *Environ. Mol. Mutagenesis* **2012**, *53*, 185–197, doi:10.1002/em.21677.
